# Acuphagia on the Obsessive-Compulsive Spectrum in an Adolescent Male

**DOI:** 10.1155/2020/8885503

**Published:** 2020-10-28

**Authors:** William Butler, Luis Allen

**Affiliations:** Department of Clinical Sciences, FSU College of Medicine, Orlando Regional Campus, 250 East Colonial Drive, Suite #200, Orlando, FL 32801, USA

## Abstract

*Background*. Pica is a condition that is commonly missed in childhood. This condition occurs worldwide and is considered normal in children from ages 18- to- 36 months. It is also commonly seen in pregnant women due to associated nutritional deficiencies. Acuphagia is a subtype of pica which has been briefly described in the literature. Its classification has been speculated to belong on a spectrum of obsessive-compulsive disorders (OCD). This case involves Mr. C, a 16-year-old male with a history of depression, anxiety, and ten previous intentional foreign body ingestions involving sharp objects such as needles, forks, and thumbtacks. He states that he recently ate a nail and denies any current obsessions. He was admitted from a local involuntary receiving facility due to decreased bowel movements in the last week. Learning points and recommendations for practitioners are described.

## 1. Case

Mr. C is a 16-year-old male with a history of depression, generalized anxiety disorder, obsessive-compulsive disorder (OCD), and ten previous intentional foreign body ingestions typically involving sharp objects such as needles, forks, and thumbtacks. He states that he recently ate a nail and denies any current obsessions. He was admitted from a local involuntary receiving facility due to decreased bowel movements in the last week. He usually stools 1-2 times per week but cannot recall the last time he stooled. He states that he has general abdominal discomfort, but it is not sharp or severe. He describes the discomfort “all over” but rubs the periumbilical areas when discussing symptoms. He otherwise denies any vomiting, diarrhea, back pain, or difficulty with urinating. He states that he had “low-grade” fevers at the outside facility. He likes to eat regular, well-varied meals. He takes Colace inconsistently and has never taken MiraLAX daily. Abdominal X-rays were performed and most recently revealed “distention of multiple segments of the colon and concern for ileus.” In addition, 16 mm metal density resembling a nail was noted to have progressed to the transverse colon. His vitals are stable (Tmax: 100.1; HR: 100 bpm; BP: 131/75 mmHg; RR: 16 bpm; O2 stats 100% in RA). He was given Tylenol 500 mg, PO, for 05/10 abdominal pain.

Of note, the patient denied any recent self-injurious behavior and denies any suicidal or homicidal ideations. He states that he is being escalated to a residential facility in another state. Previous similar episodes have required extraction by GI, including his most recent admission where he ingested a part of the IV.

He is allergic to bananas and takes the following medications: Protonix 40 mg PO qDay; Seroquel 400 mg PO qDay, Klonopin 1 mg PO at bedtime, and Prozac 20 mg PO qDay. He does not forget to take medications. His past surgical history includes three episodes of gastroscopy and esophagoscopy a year ago. His family history is noncontributory. He does not smoke tobacco products or use recreational drugs, but occasionally drinks alcohol and smokes tobacco.

His labs are within expected limits. His review of systems is positive for constipation and abdominal pain in all quadrants. On physical exam, his abdomen is soft, nontender, and nondistended, with no swelling. On this admission, the objects were passed without surgical intervention the following day, and the patient was successfully transferred back to his involuntary facility.

## 2. Discussion

This case is lends support to the literature which suggests that pica is on a spectrum of OCD related disorders [1]. However, it differs from other studies in that this patient has a type of pica termed acuphagia. This subtype involves the involuntary urge to eat sharp objects such as knives or needles. Acuphagia in someone with anxiety leading to ileus has been undocumented. This case is significant in that he reported that his behavior was an involuntary attempt to not feel anxious or depressed. He also mentioned that he was tired of being at the outside facility and hoped to be transferred to our inpatient unit (which only serves adults). Other cases which have speculated that pica is on a spectrum of OCD involve more typical features such as eating paint to soothe anxiety. Acuphagia is unique because one would expect eating sharp objects to make anxiety worse. This gives credit to the idea that all forms of pica may be on a continuum of OCD and anxiety disorders more globally.

Pica is a condition that is commonly missed in childhood [2]. The term is derived from “*Pica pica*,” which is the Latin word for brown-billed magpie. This bird eats indiscriminately, and as a result, will eat foreign objects [3]. This condition occurs worldwide and is considered normal in children from ages 18 to 36 months. It is also commonly seen in pregnant women due to associated nutritional deficiencies. However, it is uncommon in nonpregnant adults [4]. In fact, the DSM-5 explains that eating at least one or more objects for nonnutritive, nonfood substances over a period of one month is enough for a diagnosis of pica in adults [5]. In this case, the patient had ingested over 10 objects within the past year.

Acuphagia is a subtype of pica which has been briefly described in the literature. Its classification is controversial as it has been speculated to belong to a spectrum of OCD. It is worth mentioning that documented cases of acuphagia are scarce. Silverman [6] described one of the first patients in 1973. In this case, the patient was a 2-year-old girl who had a craving for metal objects. This was possibly due to a dietary deficiency, and once her lack of appetite was treated, she was cured of pica after treatment. This is the typical clinical course of pica and documented cases of eating sharp, metallic objects remains uncommon in older patients.

A more recent case by in 2007 described a 22-year-old man in Nigeria presented with vomiting after meals, cough, weakness, inability to walk, and swelling of the legs and face. The authors attribute poverty, isolation, neglect, and loneliness as triggers for his “bizarre” behavior [7]. A case from 2018 involved a young woman with a significant history of schizophrenia presenting with acuphagia [8]. She also ingested several sharp objects which were removed surgically. Our case expands upon those reported to suggest a possible linkage between acuphagia, OCD, and perhaps psychiatric illness more generally.

It is worth noting that Mr. C's acuphagia persists despite his current psychiatric treatment. He has had many inpatient visits over the past few years and is on a stable regime of medications for his depression and OCD. He has also never had a psychotic episode. The connection between OCD and other mood disorders is well studied [9]. In OCD, the behavior is typically irrational at resolving the preceding obsessions. Common themes involve handwashing, touching objects, or possibly hoarding behavior. However, a growing body of evidence demonstrates that these behaviors typically provide a sort of self-calming of internal anxieties [9]. Interestingly, this does not apply to the current case. Eating sharp objects such as nails and needles is inherently anxiety inducing. In this case, the patient's “compulsions” did not alter his underlying anxiety and depression which were being managed with psychotropic medications.

### 2.1. Guidelines Recommendations and Learning Points

Little is known about the course of acuphagia, and there are currently limited formal guidelines on the management of this disorder. Current guidelines focus exclusively on causes due to an underlying nutritional deficiency or behavioral therapy for those on the autism spectrum [10]. They emphasize a thorough assessment including a basic metabolic workup. Behavioral recommendations include environmental arrangements (e.g., locks on cabinets), teaching alternative skills (e.g., throwing the objects away), and redirection. Due to the safety risk of acuphagia specifically, these guidelines are incomplete in altering evaluation and management. These limitations warrant further investigation of this unique clinical phenomenon. Adapted guidelines for impulse control and safety suggests the following:

- Educating caregivers to arrange home environments with similar safety precautions as inpatient units (e.g., finger foods)
- Helping the client identify impulses and notifying the caregiver or sublimating with a safer activity
- Engagement in constructive activities to distract the client such as physical activity
- Enrollment in mindfulness and acceptance therapy

In conclusion, acuphagia may be a distinct psychological disorder from other forms of pica described in the DSM-5. In this case, it was unrelated to suicidal or homicidal ideations, anxiety, depression, or OCD. It has also been documented in patients with schizophrenia. While there is debate for pica to belong on a spectrum of OCD related disorders, this case suggests that the etiology may be something else. Regardless of classification, it seems to have persisted while his anxiety and depression was well controlled on psychotropic medications. Addressing nutritional needs is the first-line treatment for pica in other populations including pregnant women and children [11]. However, in our case, his nutritional needs were being met, and his mental health disorders were controlled. This suggests that acuphagia may require a unique treatment or higher level of care. Further research into the area of the appropriate treatment for this form of pica is warranted as acuphagia is a life-threatening emergency [12].
